# Recognition of a likely two phased extinction at the K-Pg boundary in Antarctica

**DOI:** 10.1038/s41598-017-16515-x

**Published:** 2017-11-24

**Authors:** Thomas S. Tobin

**Affiliations:** University of Alabama, Tuscaloosa, AL 35487 USA

## Abstract

The southernmost Cretaceous – Paleogene (K-Pg) outcrop exposure is the well-studied exposure on Seymour Island, Antarctica. Deposition across the K-Pg boundary there is uninterrupted, and as a consequence the ammonite fossil record is commonly used to test statistical methods of evaluating mass extinctions to account for the incompleteness of the fossil record. Numerous detailed fossil data sets from Seymour Island, comprised dominantly of mollusks, have been published over the last 30 years, but in most cases have not received statistical treatment. Here a previously published statistical technique is modified, automated, and applied to all published macrofossil data sets available from Seymour Island. All data sets reveal likely evidence of two separate multi-species extinctions, one synchronous with bolide impact evidence at the K-Pg boundary, and another 45 ± 15 meters (~140–290 ky) below the boundary. The apparent earlier extinction primarily affects benthic mollusks, while the boundary extinction primarily affects ammonites. While there is no unique sedimentological change over the interval where the earlier extinction is identified, it is impossible to exclude the possibility that this pattern is stratigraphically controlled. The automation of this technique allows it to be applied easily to other large fossil data sets.

## Introduction

## Cretaceous – Paleogene Extinction

The End Cretaceous, or Cretaceous – Paleogene, Mass Extinction, is the most recent of the major mass extinctions of the Phanerozoic, and has consequently drawn significant research interest. Throughout the last 30 years, a variety of hypotheses have been put forth to explain the extinction, including an asteroid impact^1^, sea level fall^2^, flood basalt eruption^3^, or some combination^4^. Recent debate has focused primarily on the relative contributions of the Chixculub impact and the Deccan Traps flood basalt eruptions^5–8^. Through modelling efforts, the Deccan Traps have been plausibly linked with environmental effects that could be extinction drivers, including acid rain^9^, sudden global cooling^10,11^, and long term global warming^12–14^. Recent work has posited a potential relationship between volcanism and impact^15^, though further research is necessary to confirm this hypothesis.

Even if direct proxy evidence could provide an incontrovertible timeline of global environmental events across the K-Pg boundary, the fossil record must be consulted to tie events with extinction, as different extinction patterns inherently support different extinction hypotheses. While few argue in support of sea level fall as a direct cause of the extinction, it is apparent that a global drop in sea level complicates efforts to examine paleontological records near the boundary by affecting stratigraphic architecture^16^. A variety of studies have argued both for^17–21^ and against^22–25^ a gradual or multi-phase extinction based on fossil data correlated with environmental proxies, and further work is necessary to resolve these differences.

There are few places around the planet with sediment outcrops containing well-preserved macrofossils without significant depositional hiatuses, but Seymour Island (SI), off the northeast of the Antarctic Peninsula appears to meet these criteria. Data from stratigraphic sections on SI has often been used to test techniques for estimating the appropriate uncertainty in the fossil record, by appending range extensions to the observed stratigraphic ranges of species, and/or by assigning confidence intervals to multi-species extinctions^26–30^. Many confidence interval methods rely on assumptions of uniform recovery potential that, while likely untrue everywhere, may be more robust on SI, particularly in the uppermost Cretaceous sediments. While not completely homogenous^31^, there is little sedimentological change across this interval in most measured sections on this island^32^ (though see Discussion).

Recently, independent data sets of molluscan fossils from SI have been used to argue for both a multi-phase^17^ and single phase^25^ extinction across the K-Pg boundary. To resolve this discrepancy, I have modified a previously published approach to estimating the confidence interval (in terms of stratigraphic width) of a multi-species extinction^27^, and applied it to every available macro-fossil data set from SI^25,33–35^. Micro-fossil data sets exist^36–38^, but they suffer from inherent problems in terms recovery potential that make them invalid for analysis here. Specifically, the assumption of a uniform recovery potential is strongly violated, because sediment samples are necessarily taken at specific intervals, and the recovery potential between the samples is zero.

## Seymour Island

**Figure 1:**
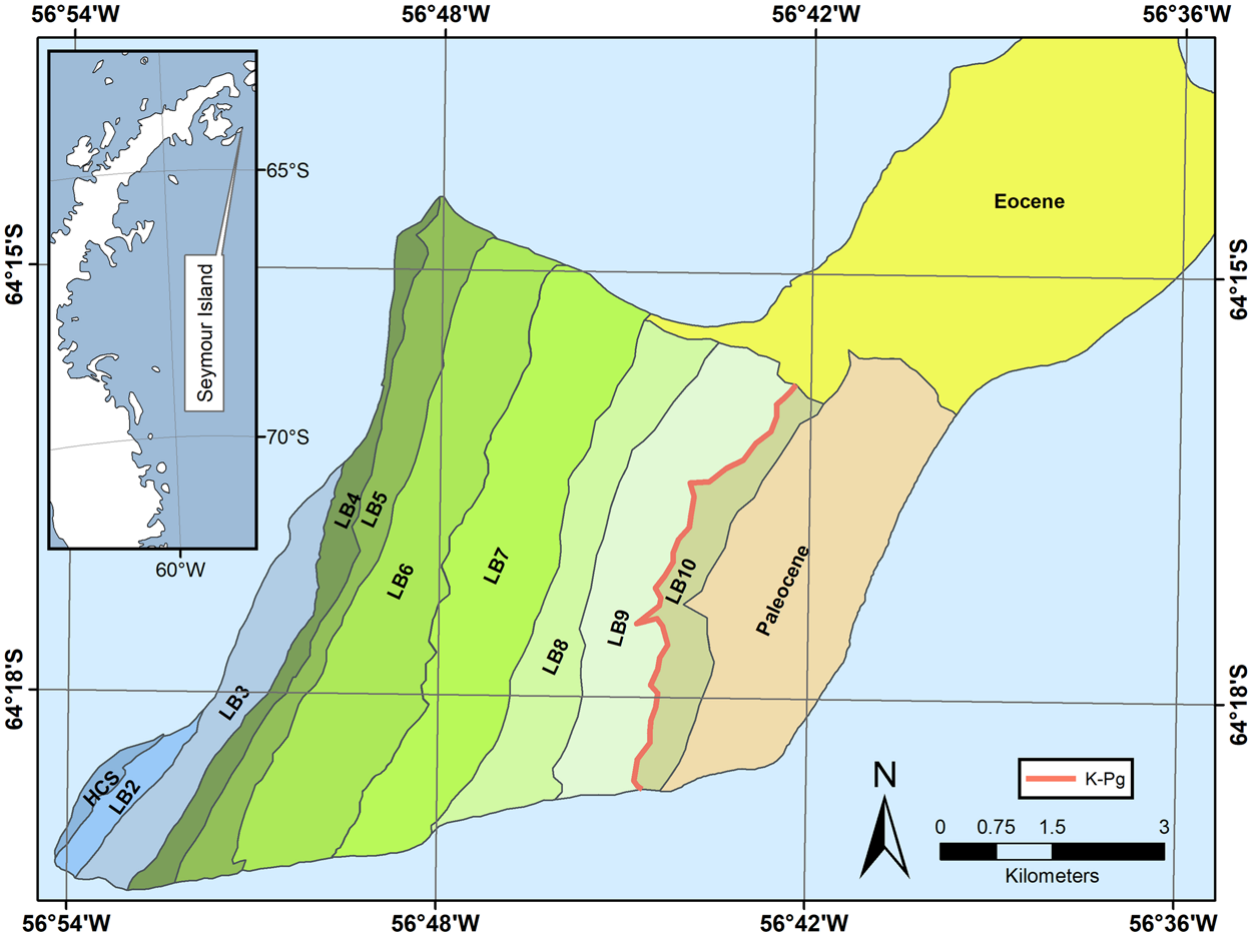
Simplified geologic map of Seymour Island, Antarctica after Macellari^32,33^ by georeferencing original figure with modification and simplification in ESRI ArcMap 10.3.1. LB designations are informal units of the López de Bertodano Formation^32^. HCS = Haslum Crag Sandstone, a recent reassignment of LB1 into a new formation^43^. Eocene and Paleocene have been greatly simplified, and more recent units have been omitted.

Seymour Island (SI) is part of the James Ross Island Group located off the northeastern tip of the Antarctic Peninsula (Fig. 1). It has experienced little tectonic activity and has not appreciably changed its latitude since deposition^17^, and the lack of alteration or significant burial has resulted in exceptional fossil preservation in terms of absolute numbers, three-dimensional morphology, and skeletal geochemistry. The basin preserves sediment from the Aptian-Albian through the Eocene, but the Maastrichtian through Danian interval on SI (López de Bertodano Formation – LBF) is probably the most well-studied due to preservation of uninterrupted deposition across the K-Pg boundary^39–43^.

Sediments of the LBF on Seymour Island are dominantly silt, with varying components of sand and clay, and have been interpreted as being deposited on an open shelf, in water depths ranging from 10’s of meters to ~200 meters^32^. Macellari^32^ divided the LBF into ten informal units in two separate groups, the lower “*Rotularia* Units” (LB2-LB6), and the upper “Molluscan Units” (LB7-LB10). The lowest unit (LB1) has since been redefined as the Haslum Crag Sandstone^43,44^. Depending on the base of section used and the particular study, this interval represents about 1100 stratigraphic meters, with the *Rotularia* Units comprising the lower ~600 meters, and the iridium anomaly representing the K-Pg boundary located at the lithologically defined LB9-LB10 boundary^45^. Molluscan fossils are much less common in the *Rotularia* Units (which are dominated by fossils of the worm *Rotularia*) than the Molluscan Units, though they are not absent. Overall, water depth increases from possibly estuarine in the lower units to fully open shelf in the upper units.

The combination of large numbers of well-preserved fossils and uninterrupted deposition makes SI an ideal place to examine paleontological records across the K-Pg boundary. Most published records have more dense fossil recovery from the Molluscan Units, which is a combination of true fossil abundance, research interest in the K-Pg extinction, and the proximity of the surface exposure of the upper units to the Base Marambio, an Argentinian outpost on the northeast part of SI used to access the island by many researchers. Because of the focus around the K-Pg boundary, logistics of base operations, and changes in deposition, it is unlikely that an assumption of uniform fossil recovery potential holds across the entire island, but that assumption may be more robust when applied to the Molluscan Units themselves. Data from this study mostly focuses on the Molluscan Units, and mostly LB9-LB10, over which there is little sedimentological variation.

## Extinction analysis

Five mostly independent fossil data sets from Seymour Island are examined using the multi-taxon confidence interval (MTCI) width pattern analysis described in detail in Methods. Briefly, the MTCI approach is based on a previously described confidence interval analysis^27^, but modifies and expands on it to calculate the stratigraphic width of an extinction confidence interval at every possible stratigraphic level, rather than at arbitrarily chosen points. It is important to note that the earlier approach^27^ assumes an extinction exists and finds an MTCI width assessing the certainty of its location. Here, this method is instead employed as a tool to find intervals where last occurrence data (LAD) (incorporating range extensions) are condensed, meaning the MTCI width is effectively a measure of how condensed or synchronous the LAD are. Unusually condensed intervals, as compared with randomized fossil data sets, are interpreted as possible extinctions.

A collection of MATLAB scripts (available as supplementary documents) were written to automate this process, which ultimately generates an MTCI width at the stratigraphic level of every LAD in the fossil data set. The script then compares the generated width pattern from fossil data to patterns generated from randomized fossil data sets that share the same basic parameters as the fossil data. Confidence intervals widths from fossil data that were narrower than the 2.5% percentile from randomized data sets were considered likely intervals of multi-species extinction (though see discussion of stratigraphic control below). Unless otherwise stated, these data sets are all analyzed using a 95% confidence threshold for the MTCI and 100 random comparison trials (see Methods). All figures display individual taxon range extensions of 25% for comparative reasons, but range extensions are varied in the analysis to minimize MTCI width at each stratigraphic height.

Throughout, taxa colored in purple represent presumably free swimming organisms, while taxa colored green represent obligate benthic groups. Each taxa is numbered, and the specific taxonomic designations can be found in the supplemental material. While all data are from SI, most use different stratigraphic section bases, or are collected from different parts of the island and therefore have different measured stratigraphic heights for the K-Pg boundary. Stable isotopic data suggest that using the K-Pg boundary as a datum is a reasonably effective correlative tool, especially within the Molluscan Units^46^. In all cases, the stratigraphic height of the K-Pg boundary is known from abiotic evidence, and indicated by a horizontal red line in figures. While known, the boundary location is not utilized in any calculations of extinction placement. The data is described in chronological order of publication, starting with the earliest.

## Results

### Macellari (1986)

This data set is the earliest available that provides stratigraphic information on individual taxonomic occurrences, allowing the MTCI width pattern analysis described above (and in Methods) to be attempted. Macellari^33^ published information only on ammonites, and is therefore one of the smallest data sets (143 horizon occurrences) analyzed here, and also suffers from a lack of comparative survivors, as all ammonites went extinct at the K-Pg boundary. This data has been used previously in assessing the rapidity of the K-Pg mass extinction^26,28,29^, generally with the conclusion that the extinction of ammonites on SI was consistent with a sudden rapid extinction like that caused by a bolide impact. Stratigraphic data was obtained by digitizing Fig. 5 from Macellari^33^ using freely available open source software^47^ as described in Methods.

**Figure 2:**
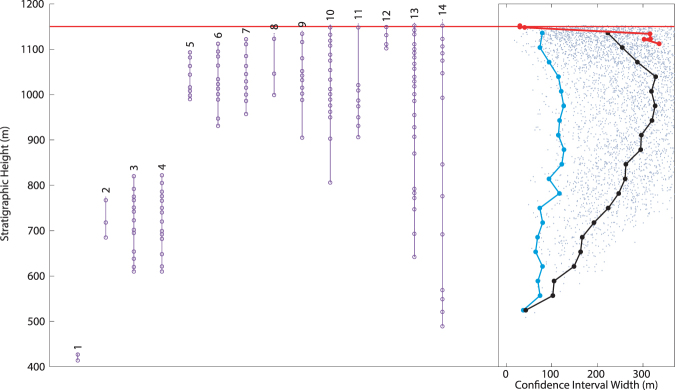
Taxonomic ranges and MTCI with pattern from data from Macellari^33^. Red horizontal line indicates the location of the K-Pg boundary. At right, grey dots: MTCI width data from randomized trials; black line: median of the randomized data; blue line: 2.5 percentile line; red line: MTCI width pattern from the fossil data. Pattern is consistent with a single extinction simultaneous with the K-Pg boundary.

Figure 2 reveals a pattern consistent with a single sudden extinction at the K-Pg boundary, though this interpretation may be complicated by the relatively small size of the data set (compared to those described below), which necessitated using 500 background randomized data sets, instead of the usual 100. The MTCI width pattern is smaller than the 2.5% percentile window only at the K-Pg boundary, indicative of a single phase extinction for ammonites, as found in previous studies. Because this data includes no surviving taxa, the extinction interval identified here is difficult to separate from edge effects (see Methods) that can cause an artificial extinction at the top of a measured stratigraphic range. However, in this case the LAD of individual taxa are not artificially terminated, but represent a failure to locate specimens over the next 100 meters, and therefore interpreting this interval as a potential extinction is appropriate.

### Zinsmeister *et al*. (1989)

**Figure 3:**
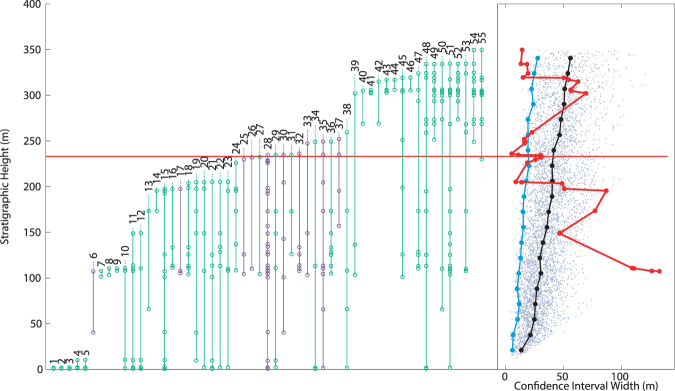
Taxonomic ranges and MTCI with pattern from data from Zinsmeister *et al*.^34^. Red horizontal line indicates the location of the K-Pg boundary. Green taxonomic data are benthic organisms, purple are free-swimming. At right, grey dots: MTCI width data from randomized trials; black line: median of the randomized data; blue line: 2.5 percentile line; red line: MTCI width pattern from the fossil data. Pattern is consistent with a two phase extinction, one 30 meters below the boundary, the other simultaneous with the K-Pg boundary.

Data from Zinsmeister *et al*.^34^ includes the largest variety of macrofauna, including five groups of mollusks, as well as worms, arthropods, and marine reptiles. While the overall number of fossil occurrences is smaller than some other data sets (326 horizon occurrences), the stratigraphic focus around the boundary is narrower, and the fossil density comparable over this interval to others. Data were obtained by digitizing Fig. 3 in the original publication^34^, and hand selecting fossil occurrences using freely available open source software^47^. While studies by Zinsmeister have included fossil localities from all over the island, this data set was selected because it was restricted to a single stratigraphic section that did not rely on stratigraphic plane projection for correlation across the island^48^. Fossil data were used as presented in the original manuscript, which includes location of some ammonites in the Paleogene, which have been interpreted as reworked. This mixed macrofossil data set indicates two separate extinctions (Fig. 3), one almost simultaneous with the boundary, and another 31 meters below the first, broadly consistent with a more rudimentary confidence interval approach applied to this data^17^. The narrow MTCI width at the top of the section here is the consequence of artificial range truncations (see Methods, edge effects). The previously observed selectivity of the extinction was also preserved: the lower extinction primarily affects benthic organisms, while the boundary associated extinction leads to the extinction of most free swimming organisms (primarily ammonites) in the data set. A second analysis of the data was attempted after removal of one marine reptile occurrence and six ammonite occurrences from five taxa and that were considered reworked. The observation of two extinctions over this interval, and particularly the presence of the lower extinction, was confirmed, though the separation between the two events was less clear (Fig. S1).

### Stilwell *et al*. (2004)

**Figure 4:**
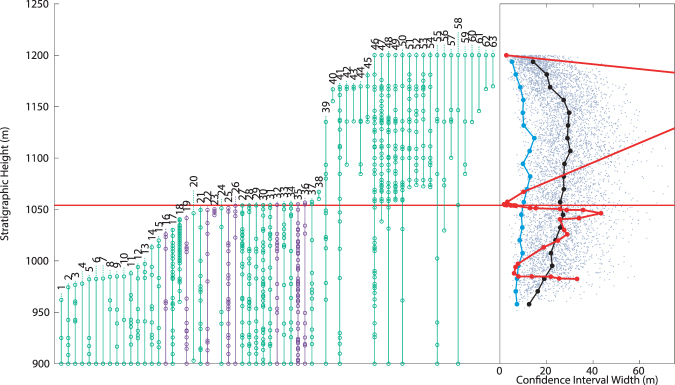
Taxonomic ranges and MTCI with pattern from data from Stilwell *et al*.^35^. Red horizontal line indicates the location of the K-Pg boundary. Green taxonomic data are benthic organisms, purple are free-swimming. At right, grey dots: MTCI width data from randomized trials; black line: median of the randomized data; blue line: 2.5 percentile line; red line: MTCI width pattern from the fossil data. Pattern is consistent with a two phase extinction, one 66 meters below the boundary, the other simultaneous with the K-Pg boundary. Artificial range truncations at top of range generate an artificial extinction at 1200 meters.

This study focused on examining molluscan recovery after the K-Pg extinction, but presents a detailed (702 horizon occurrences), albeit stratigraphically short, record in the latest Cretaceous. Data were obtained through digitization of Fig. 2 in the original manuscript^35^. It should be noted that many of the taxa in this interval are indicated as ranging either below the lower boundary or above the highest boundary of the figured stratigraphy, without specific occurrence data provided. In these situations, an additional single occurrence for any taxa ranging out of the figure was indicated at graph boundaries. It is important to note that this data is only partially independent from that of Zinsmeister *et al*.^34^, specifically, some of the Cretaceous molluscan fossil data is shared between the two data sets (Jeffrey Stilwell, personal communication). The 1989 data set includes a variety of non-molluscan fauna, and ranges lower in section, while the 2004 data set includes a significant amount of new fossil data unavailable in 1989 and ranges higher in section. Analysis of this data reveals two separate extinctions, one simultaneous with the K-Pg boundary, the other 66 meters below (Fig. 4). The selectivity of these extinctions, in terms of benthic and free-swimming organisms, follows the same pattern as in the analysis of Zinsmeister *et al*.^34^.

### Witts *et al*. (2016)

The most recently published study of SI molluscan extinction argued for a single sudden extinction at the K-Pg boundary^25^. This study combined multiple different data sets to construct ranges of taxa across SI from three different stratigraphic sections measured and sampled in 1999 (Section A), 2006 (Section B), and 2010 (Section C), as well as some previous published data^49^. An important element in calculating the length of individual taxon range extensions is the number of horizons at which fossil occurrences are recorded, and a composite data set renders this approach untenable by potentially artificially inflating the number of horizons. Fortunately, supplemental appendices from Witts *et al*.^25^ separate the data from the independent stratigraphic sections, and provide detailed range and occurrence for taxa within each. Here Sections A and B are analyzed, as Sections C and that from Zinsmeister *et al*.^49^ are too stratigraphically short (80 and 18 meters respectively) to provide a useful record.

**Figure 5:**
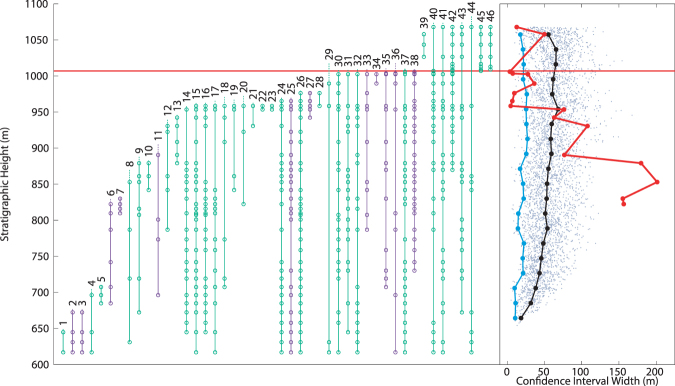
Taxonomic ranges and MTCI with pattern from data from Section A from Witts *et al*.^25^. Red horizontal line indicates the location of the K-Pg boundary. Green taxonomic data are benthic organisms, purple are free-swimming. At right, grey dots: MTCI width data from randomized trials; black line: median of the randomized data; blue line: 2.5 percentile line; red line: MTCI width pattern from the fossil data. Pattern is consistent with a two phase extinction, one 31–49 meters below the boundary, the other simultaneous with the K-Pg boundary. Artificial range truncations at top of range generate an artificial extinction at 1068 meters.

**Figure 6:**
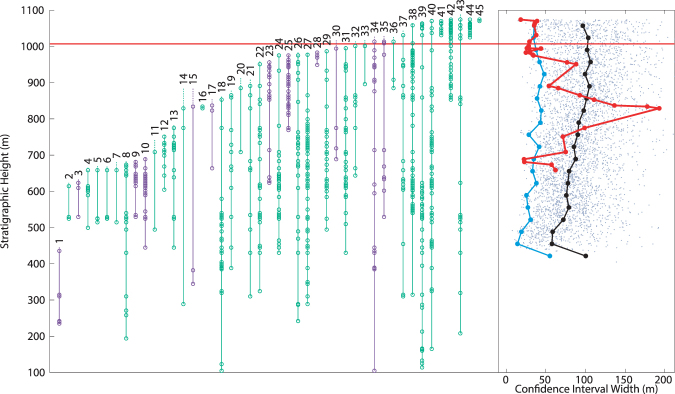
Taxonomic ranges and MTCI with pattern from data from Section B of Witts *et al*.^25^. Red horizontal line indicates the location of the K-Pg boundary. Green taxonomic data are benthic organisms, purple are free-swimming. At right, grey dots: MTCI width data from randomized trials; black line: median of the randomized data; blue line: 2.5 percentile line; red line: MTCI width pattern from the fossil data. Pattern is consistent with a two phase extinction or a single extended period of extinction starting 32 meters below the K-Pg boundary and extending until just above the boundary. Artificial range truncations at top of range generate an artificial extinction at 1074 meters.

Section A, collected in 1999, includes 495 fossil horizon occurrences over ~500 meters of stratigraphic section. MTCI width pattern analysis of this data indicates two separate extinctions, one simultaneous with the boundary, the second 31–49 meters below (Fig. 5). Interpretation of the placement of the lower extinction is complicated because the MTCI widths do not have a single clear minimum, instead a range of comparable minima over 18 meters. However, the lower extinction is clearly distinct from the boundary extinction. Additionally, the lower extinction is largely comprised of benthic mollusks; as in above analyses, the ammonites largely persist until the K-Pg boundary. Section B, collected in 2006, includes 659 fossil horizon occurrences over ~1000 meters of stratigraphic section. Analysis of this section provides the most equivocal MTCI width pattern examined here (Fig. 6). A possible extinction is recorded 326 meters below the boundary, below the analyzable stratigraphic range of the records above, and at an interval that may be associated with a facies change (see Discussion). A second extinction spans the K-Pg boundary, but may reach as low as 30 meters below the boundary. There is some suggestion that this interval would be more appropriately represented as two separate events, due to the widening of the MTCI width pattern 13 meters below the boundary.

## Discussion

### Common patterns

Application of the MTCI width pattern analysis developed here reveals some consistent patterns between data sets. With one exception, data sets that incorporate benthic mollusks show two clear intervals where LAD are unusually condensed, one simultaneous with the K-Pg boundary, the other 45 ± 15 meters below the boundary. The only exception (Section B from Witts *et al*. 2016), shows a pattern that is plausibly consistent with either a single or double event. The difference in stratigraphic height of this earlier event is likely due to small variations in the thickness of different units across the island^32^ though overall the units of the LBF are approximately tabular^31^. A prior similar observation^17^ was interpreted as representing two separate extinctions, the second related to the K-Pg bolide impact, and the first of which was linked with the onset Deccan Traps flood volcanism, based on a temporal link through magnetostratigraphy and a link to an increase in local seawater temperatures^17,21^. The temperature increase in the Cretaceous portion of C29R has been observed globally^50–52^, and the contemporaneity of the two has suggested a connection with the onset of Deccan Traps. The selectivity of the lower extinction for benthic mollusks and the upper for free swimming organisms would add circumstantial support for different causes of the extinctions. If this interpretation is correct, it is possible the lower extinction is a global event that is only observed on SI due to the expanded nature of the section (0.1–0.2 mm/a), but the early extinction on SI could also be explained by a local or regional event. Seymour Island was located at a high paleolatitude location during the K-Pg interval, and if the early extinction observed here is real and climate related, it may be due to the increased sensitivity of polar regions to temperature change.

### Potential Stratigraphic control

**Figure 7:**
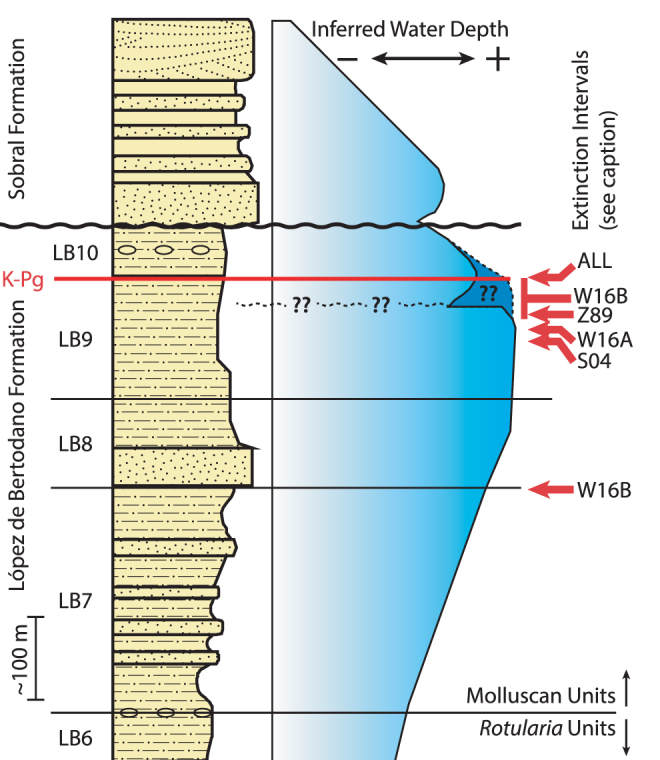
Approximate placement of identified extinction points from different data sets on a composite stratigraphic section and inferred water depth curve after Olivero^43^ and Macellari^32^ respectively. All (ALL) groups show an extinction synchronous with the K-Pg boundary, and data sets that include benthic organisms indicate an earlier extinction 30–60 meters below the boundary. Datasets: W16A – Witts *et al*.^25^ Section A; W16B – Witts *et al*.^25^ Section B; Z89 – Zinsmeister *et al*.^34^, S04 – Stilwell *et al*.^35^. The shallowing and possible disconformity plotted at 30 meters below the boundary was inferred largely from paleontological patterns by Macellari^32^, though Crame *et al*.^53^ and the author disagree with this interpretation, favoring a pattern depicted by the darker blue and dotted line (see discussion).

Previous studies of SI suggest another possible explanation for the early extinction of benthic mollusks. Macellari^32^ “tentatively proposed” a sudden regression, and possible minor depositional hiatus 25–30 meters below the boundary (Fig. 7). If this regression, or more accurately a rapid shallowing, occurs stratigraphically just above the lower extinctions observed in several macrofossil data sets, it could imply a depositional control on species ranges instead of a true extinction at this level. Evidence for this regression is based primarily on the faunal changeover at this point, though this horizon is also marked by somewhat elevated bioturbation and presence of intraclasts in from on locality in one out of the five boundary crossing sections measured by Macellari^32^. This evidence could indicate a condensed section from a sea level high stand, during which sedimentation would slow and burrowed hardgrounds can develop. The author has not visited Section D of Macellari^32^, where the intraclasts were observed, but has examined Sections F and C without noting any unique sedimentological changes over the 100 m below the K-Pg boundary at either location. Similarly, Crame *et al*.^53^ measured and logged section across the approximate location of Section D from Macellari^32^ and state that they find “no clear evidence … for the stratigraphic hiatus reported by Macellari.”

With no discernable sedimentological changes in the interval prior to the K-Pg boundary, the interpreted shallowing 30 m below the boundary is defined exclusively on fossil evidence, and a tautology emerges in determining whether the early extinction is a real event or the consequence of a subtle facies control. The paleobiological record itself shows that some taxa that persist through the early extinction have gaps in their range record at this interval, most notably 8 of 19 surviving taxa from Witts *et al*. Section A (Fig. 5). Gaps of this nature have been suggested to result from facies changes^54^, which could lend support to a stratigraphic explanation for the potential extinction pattern observed. The lack of clear sedimentological change over this interval does not rule out a stratigraphic control on the fossil patterns observed above, and further detailed sedimentological research could test this hypothesis.

Regardless of whether the potential early extinction is biological or not, it is clear that the method employed here is sensitive to stratigraphic controls where sedimentological changes are observed. The lowest potential extinction (326 meters below the K-Pg), only observed in the data from Section B of Witts *et al*.^25^, is associated with a change in the substrate from mostly silt to a significantly sandier interval, demonstrating the importance of facies or stratigraphic controls. Due to this sedimentological change, it would be inappropriate to interpret the condensed LAD pattern at this level as an extinction. In fact, a similar condition may affect the potential extinction identified at the K-Pg boundary. The K-Pg interval was recognized as a distinct sedimentological change between Units 9 and 10 of the LBF prior to the recognition that it contained the K-Pg boundary. This well-cemented and glauconite rich layer could indicate a condensed section, possibly making the K-Pg extinction appear more abrupt than it was. However, given the broader context of this event and evidence for bolide impact, a sudden extinction at this point is the most likely interpretation.

## Conclusion

Ultimately, while the molluscan data sets examined here provide largely consistent records, the paleobiological data itself is unable to unequivocally resolve whether the apparent early extinction is an artifact of sequence stratigraphic control on the fossil record or a real event^16^. However, without a clear and unique sedimentological indication of facies change over the last 100 meters of the Cretaceous, a true biological event, rather than stratigraphic control, is better supported by the paleobiological evidence. This result supports the conclusions of previous work^17^ and those that have relied on it^21^ that there are two separate extinction events, one prior to the K-Pg boundary, and one simultaneous with the bolide impact at the K-Pg boundary. This earlier extinction is occurs approximately 200 ka prior (66.2 Ma) to the K-Pg boundary, based on assumptions of depositional rate from magnetostratigraphy^17^. Work here presents yet more evidence that detailed studies of extinction rely heavily on carefully constructed sedimentological and paleobiological records, and that is impossible to divorce the fossil data from their geologic context.

## Methods

Ascertaining whether a mass extinction is a single event, a gradual decline, or a multi-phased scenario is challenging. Wang and Everson^55^ developed a technique for examining pulsed extinctions, but it requires *a priori* knowledge about which species are included in which pulse. Similarly, other methods for ascertaining the width of a confidence interval of a mass extinction require some determination of which species to include in the analysis^27,29^. Here the confidence interval approach of Wang and Marshall^27^ is modified and expanded upon to examine all possible combinations of extinction intervals, using increasingly greater numbers of species. Extinction events are identified as confidence interval widths that are narrower than those generated by random simulations of the same size data set. It does not require an initial determination of which species subject to the extinction or extinctions, and is capable of recognizing multiple pulses of extinction. The method of Wang and Marshall^27^ as deployed here does require an assumption of uniform recovery potential due to the use of range extensions as described by Strauss and Sadler^56^, which many others have made for Seymour Island, but this does mean that stratigraphic controls could influence the results (see Discussion). This modified method has been largely automated for properly formatted fossil data using MATLAB scripts available in the online supplemental material. These scripts also generate stratigraphic range plots of fossil data, sorted using their original order or by last occurrence.

It is first necessary to clarify how the method employed here utilizes and modifies previously published techniques^27^. First, individual taxon range extensions (*r*_*c,i*_) are calculated based on number of fossil horizons (*H*), total species range (*R*), and desired confidence level (*C*, 0–1), which relies ultimately on the equation (1) developed by Strauss and Sadler^56^:

1 $${r}_{c,i}=[{(1-C)}^{-1/(H-1)}-1]R$$

This calculation is insensitive to multiple occurrences of the same taxon from the same horizon. One of the novel observations of Wang and Marshall^27^ and Marshall^26^ was that these single taxon range extensions could be combined to generate a multi-taxon confidence interval (MTCI) on the location of a multi-taxon extinction event, and further, that despite utilizing relatively low confidence (<50%) range extensions (*r*_*c,i*_), a st*r*atigraphically well-constrained, high confidence MTCIs could achieved. A binomial distribution is used to calculate the probability that the true extinction horizon lies between any given end points of individual taxon range extensions. A higher confidence is achieved by including a greater number of taxa, at the expense of expanding the stratigraphic (or temporal) width of the interval. Further they posited the highest fossil occurrence could be used as a base to the MTCI, below which the extinction could not have occurred.

The width of the MTCI is effectively a measure of how close the LAD of multiple taxa are to each other, and how many taxa are included in the analysis. Smaller MTCI widths indicate some combination of larger number of taxa and a high degree of contemporaneity of LAD. This implementation is a subtly different use of this method than that intended by Wang and Marshall^27^. They intended this technique to assume a sudden extinction existed, and then measure how certain they could of its location. In effect, narrow stratigraphic MTCI widths occur when the LAD (accounting for individual taxon range extensions) are more synchronous. These MTCI widths are then assessed against randomized fossil data sets, and intervals where the LAD are unusually condensed are interpreted as extinctions, as explained more fully below.

The MTCI method is applied to a fossil data set in a stepwise fashion, by analyzing increasing numbers of taxa, which are sorted initially by last appearance datum (LAD). While a commented code for this process is available in the supplementary material (ConfInt.m), it is described qualitatively in this section. It should be noted that any singleton taxa are removed from the analysis as their range extensions cannot be calculated. Starting with the first two taxa (lowest two LAD), an attempt is made to generate a MTCI that is above a prescribed threshold (e.g. 95%), though this threshold can be varied within the code (see below for effects). The script then analyzes first three taxa, and so on until all taxa are included. In situations where multiple taxa have the same exact LAD, then all of them are added to the analysis at the same time. For each group of *n* taxa, a range of *C* values are tested such that each *C* value is some multiple of 1/*n*, up to and including 0.75. Starting with the lowest *C* value, an attempt is made to construct the stratigraphically narrowest possible MTCI that has a confidence value greater than the confidence threshold. If a valid MTCI is identified, it must necessarily be narrowest possible MTCI for this group of taxa, and information about the width of the confidence interval is stored, and analysis continues incorporating an additional taxon. If no valid MTCI is obtained, the next highest *C* value is attempted, until an MTCI is located or no *C* values remain, in which case a NaN value is stored and the next group of taxa is analyzed. The placement of this point is at the highest LAD analyzed, which is not necessarily the exact point of a multi-taxon extinction, which could range slightly higher in the stratigraphy. In such a way, the narrowest possible MTCI at each horizon is calculated, and a pattern can be produced plotting the width of the MTCI through the stratigraphy (red line on right side of Figs 2–6). The highest LAD is not utilized as the base of the MTCI, as it can create a false narrowing of the MTCI that is best avoided here (see below for more). This process is performed by the script multispec.m, a subroutine of ConfInt.m.

To better interpret the MTCI width pattern, 100 randomized taxonomic data sets are generated for comparison, using the same number of taxa and total number of fossil occurrences (after removal of singleton taxa), but with the number of occurrences per taxon and their stratigraphic heights randomized. After the script user selects a value of *C* (also used for calculating *r*_*c,i*_ for display purposes), the number of taxa with *r*_*c,i*_ overlapping the highest LAD are used to calculate a survival percentage. This survival percentage (randomized ± 10%) was applied to taxa in the random data sets, which were allowed to occur throughout the investigated stratigraphic range, while the rest had randomized range terminations (the effects of this decision are explored below). An MTCI width pattern is generated for each randomized data set, which are plotted alongside the MTCI width pattern from the true fossil data. These MTCI width data points are then treated as a single data set, and separated into a user-defined number of stratigraphic bins. For each bin, the median and 2.5% percentiles are calculated and plotted (grey data on right side of Figs 2–6). Intervals where the fossil data MTCI width pattern is smaller than the 2.5% percentile represent intervals where the LAD are much more synchronous than any expected by random chance, and thus indicate a probable multi-taxon extinction event.

Attempts to perform this analysis with microfossils were complicated by the inherent limitations of microfossil collection and identification. The microfossil record is represented by discrete sediment sampling locations, with a 0% recovery chance in between sampling sites. For the analysis used here, this generates intervals of high extinction intensity at many sampling levels that are likely artificial, particularly when sampling is less intense.

### Parameter effects

Several parameters can be adjusted in this approach that may affect both the generation of the MTCI pattern from fossil data, but also the distribution of random points for comparison. Ultimately, none of these values have effects that would change the interpretation of the record (except at extreme values), implying the overall approach is robust.

#### Confidence threshold

When generating a MTCI for each group of taxa, a minimum MTCI width is determined for a given percentage threshold, which is set at 95% for analyses presented here. Changing this value does affect the shape of the MTCI width pattern, and also affects the distribution of random comparison data (since they are generated using the same approach and subscript – multispec.m). In general lower confidence thresholds reveal more texture in the fossil data, but make real extinction intervals harder to discern. In addition lower confidence thresholds can lower random MTCI width patterns so much that there is no ability to discern any extinction intervals from background data (Fig. S2) as many widths begin to approach zero when the confidence threshold is lowered below 80%. A confidence threshold of 95% appears to balance these competing factors well for all the examined data sets, and is employed here throughout.

#### Use of last LAD

This script does not use the highest LAD within the analyzed taxa as the base of the MTCI as suggested in the original publication^27^, because to do so can create artificially short MTCI widths. The MTCI width in the analysis used here is effectively a proxy for how synchronous the LAD of multiple taxa are when accounting for *r*_*c,I*_, and using the highest fossil LAD as the base removes this relationship. This effect is is clearly shown when examining real fossil data^35^. Figure S3 can be compared directly with Fig. 4, where two clear intervals of extinctions are shown. When the highest LAD is used (Fig. S3), both the actual data and the random data collapse towards zero for the MTCI width, which renders any patterns in the data undecipherable. This observation holds for all the fossil data sets examined above.

#### Randomized data

MTCI width patterns are generated for a number of randomized data sets based on the size and distribution of the original fossil data (see above). All of the data points from those randomized MTCI width patterns are treated as a single data set, and binned into stratigraphic windows. If sufficiently large numbers of randomized data sets are employed (generally >50), the number of bins used has a negligible effect on the calculated percentile widths (Fig. S4). The appropriate number of bins will be dependent on the number of fossils in the data set and the stratigraphic range over which they’re distributed, but the exact number will have little effect on the conclusions. Similarly, the total number of randomized data sets in the comparison data does not have a substantial effect unless a very small number are used. For all the analyses on fossil data, 100 randomized data sets were used except in one case. Future users are encouraged to explore the effects of different numbers of randomized data sets to ensure they are using a sufficient number.

#### Edge effects

Noticeable effects can occur near the tops and bottoms of the analyzed stratigraphic intervals that are worthy of discussion. With the true fossil data, very narrow MTCI widths occur if LADs for surviving species are all very close together for artificial reasons, creating an apparent, but false, extinction at the end of the examined stratigraphic interval. This effect is particularly pronounced if range through taxa have their final occurrence mapped as the top of the interval, as was modelled for data from Stilwell *et al*.^35^ (Fig. 4). The knowledge that the area above the identified extinction interval has been searched unsuccessfully for specimens of the included taxa would indicate that these LAD are not artificial, and therefore the MTCI width pattern can be interpreted appropriately. This is most notable when surviving taxa are included in the analysis, but in the case of an ammonite only data set^33^, it is known that the overlying interval was examined carefully.

A narrowing of random comparison data often occurs at the bottom of the examined stratigraphic interval, though this effect is unrelated to the above issue. At the low end of the stratigraphic interval, only a small number of taxa are included in the MTCI calculations. In most cases, this means that there is no viable MTCI that passes the 95% confidence threshold with a small number of species, and no width is calculated. However, with a reasonable number of random comparisons, a small number of cases will have a higher number of taxa simulated as going extinct early, and they will necessarily generate a very narrow MTCI, because their LADs will be spread over a narrow stratigraphic range. Additionally, because each taxon has a randomized number of occurrences, these simulated short lived taxa will often have high sample recovery and therefore very short range extensions, which contributes to a narrow MTCI. This pattern is observable to some extent in the randomized comparison points in all examined data sets (Figs 2–6). Both the upper and lower narrowing effects place limits on the use of the technique employed here. The examined extinction boundary should ideally be somewhat removed from both the lower and upper bounds of sampling, so that sufficient “background” extinction rates are available for comparison.

#### Digitization of figures

For some of the analyzed data sources, particularly older publications, no original data tables survive. In these cases, figures with occurrence and range data were digitized with freely available software^47^ with modelled axes using stratigraphy and taxa (numbered sequentially). Fossil occurrences were then hand-picked, and data was processed so that taxa were all represented by whole numbers. Occasional errors may occur during the digitization process when the visual depictions of fossil occurrences are dense and overlap, leading to an underestimate of total occurrences, and hence an overestimate of appropriate range extensions. The consequence of any extended range extensions would be to slightly extend MTCI widths, so this approach would possibly hide an extinction and should be considered conservative.

An additional issue that occurs with digitization is a small amount of artificial stratigraphic noise that is introduced, though generally less than 1% of the stratigraphic position. The effect of this error is negligible in calculation of individual taxon range extensions or MTCI widths, but can, in certain situations, affect the number of points in used in generating an MTCI. The script adds taxa incrementally, sorted by last occurrence, but when simultaneous last occurrences are present, all taxa with that last occurrence are added simultaneously, resulting in a single point for that stratigraphic height. With small amounts of noise, the taxa will be added one by one, which increases the number of heights at which the MTCI width is calculated. However, this generally does not affect the overall shape of the MTCI width pattern. Figure S5 shows the effects of adding a random amount of noise to all fossil occurrences in data that is otherwise set at fixed stratigraphic heights. Identified extinction intervals and overall pattern are the same in both, as is the case in all other digitized scenarios tested.

## Electronic supplementary material

Supplementary Figures and Scripts

Supplementary Dataset 1
