# Supplementary material for: Recognition of a likely two phased extinction at the K-Pg boundary in Antarctica
Source: Sci Rep. 2017 Nov 24;7:16317. doi: 10.1038/s41598-017-16515-x (PMC5701184; doi:10.1038/s41598-017-16515-x)
Supplement: Supplementary file 1 — Supplementary Figures and Scripts [file 41598_2017_16515_MOESM1_ESM.pdf]

Supplementary information for: "Recognition of a likely two phased extinction at the K-Pg boundary in Antarctica"

Thomas Tobin* - University of Alabama, ttobin@ua.edu

Supplemental Figures

Supplemental Figure 1. Taxonomic ranges and MTCI with pattern from data from Zinsmeister (1989) with likely reworked samples removed. Red horizontal line indicates the location of the K-Pg boundary. Green taxonomic data are benthic organisms, purple are free-swimming. At right, grey dots: MTCI width data from randomized trials; black line: median of the randomized data; blue line: 2.5 percentile line; red line: MTCI width pattern from the fossil data.

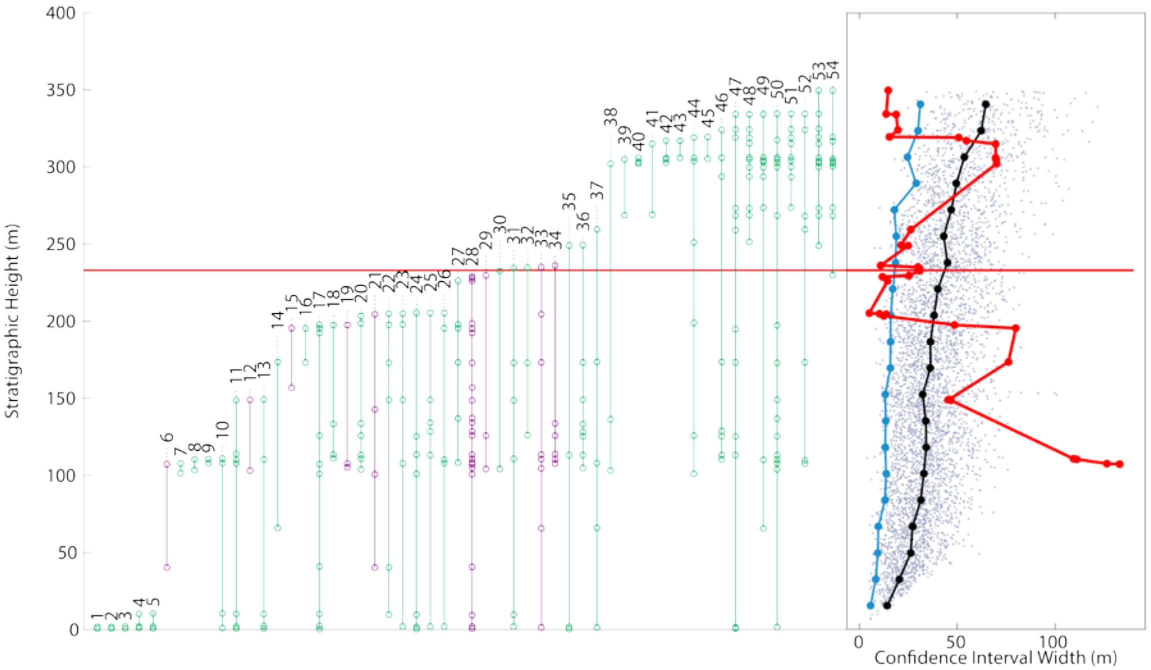

Supplemental Figure 2. Comparison of changing confidence thresholds on MTCI width pattern using *C* = 80%, 90%, 95%, 99% (100 trial analyses). Data tested from Witts et al. 2016, Section A.

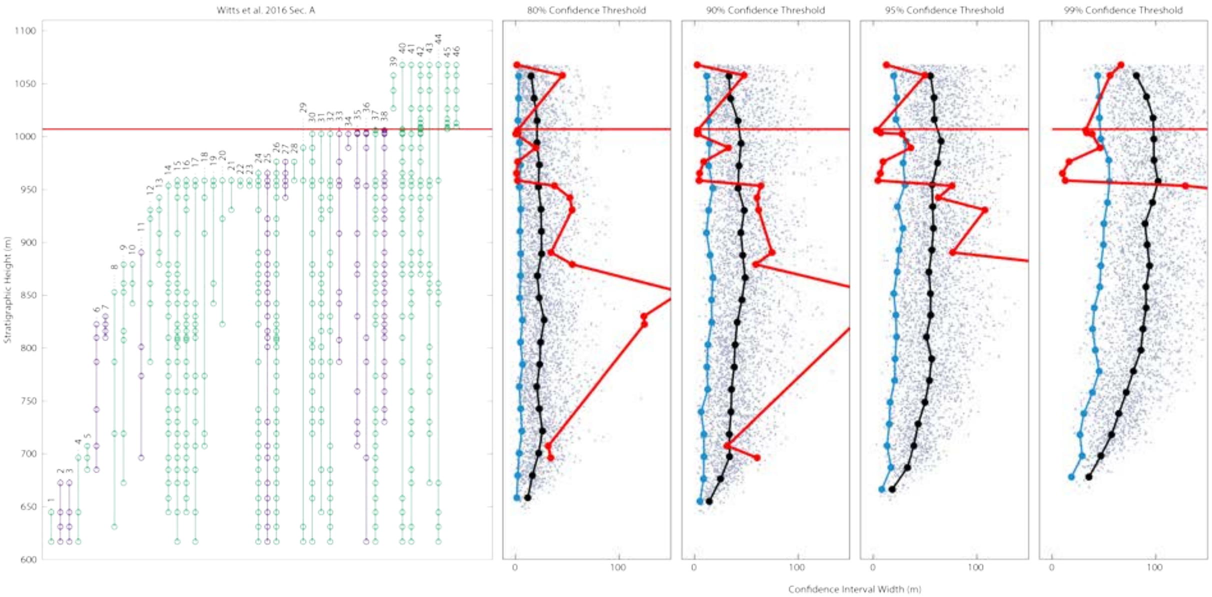

Supplemental Figure 3. MTCI width pattern analysis of data from Stilwell et al. 2004, using the highest fossil occurrence as the base of the MTCI. Compare with figure 4 in main text.

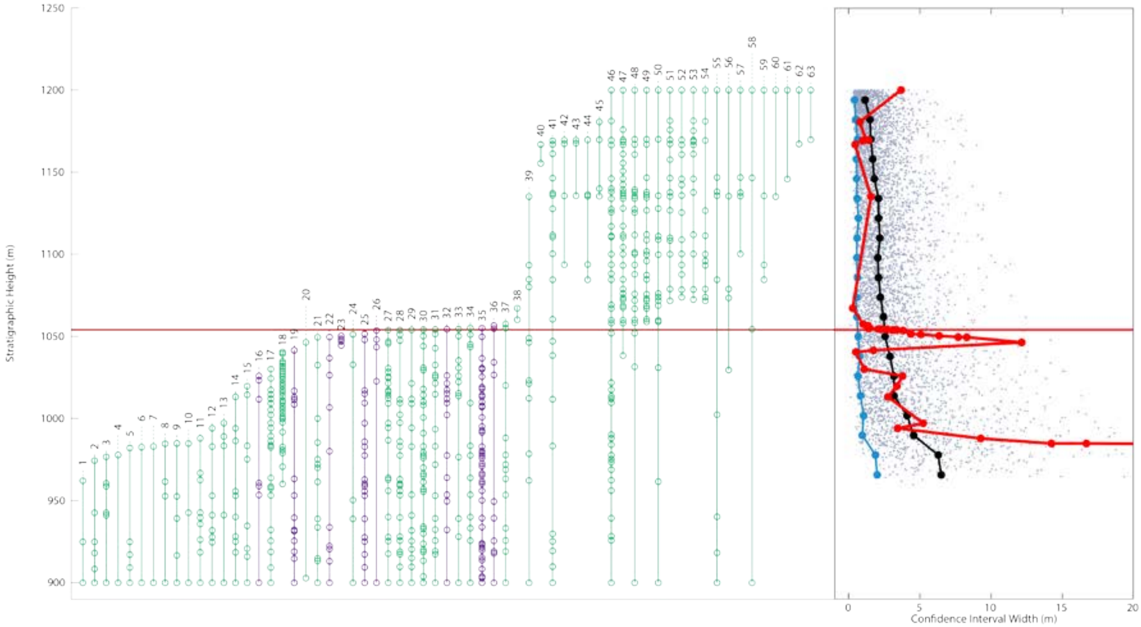

Supplemental Figure 4. Comparison of the use of stratigraphic binning when calculating 2.5% percentile and median values for randomized data using data from Witts et al. 2016, section B. There is no important difference in how the fossil data compares with randomized data regardless of how many bins are used.

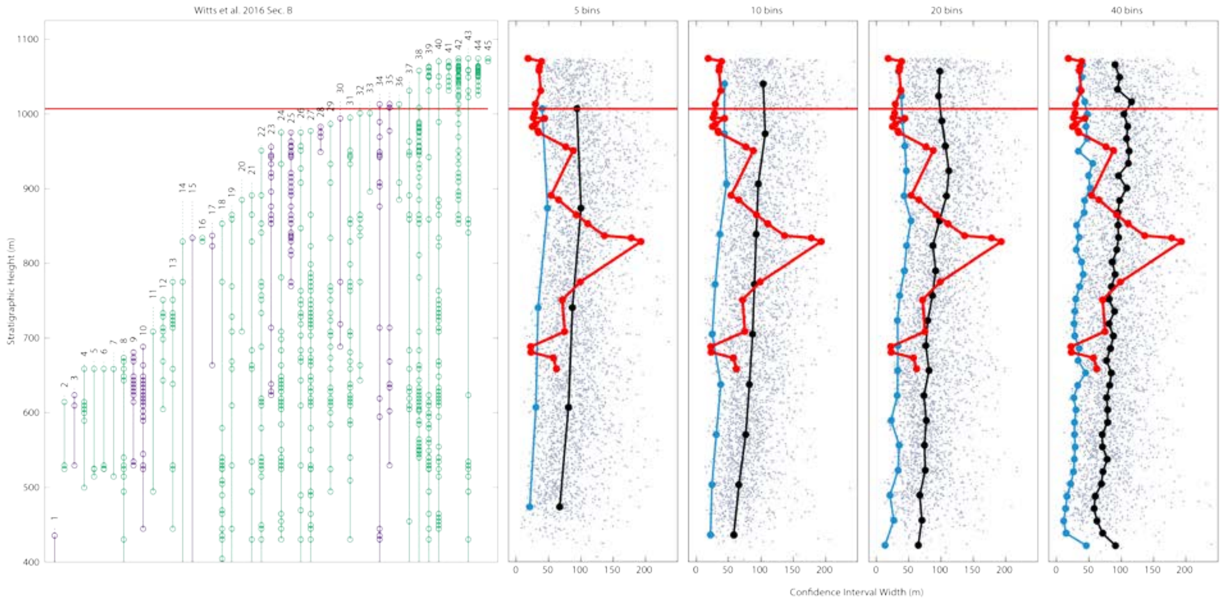

Supplemental Figure 5. Test of potential errors derived from figure digitization using data from Zinsmeister et al. (1989). Random noise was added to each stratigraphic point in the data set within the ranges in subfigure titles. No major changes in pattern were observed even for ranges well outside reasonable digitization errors.

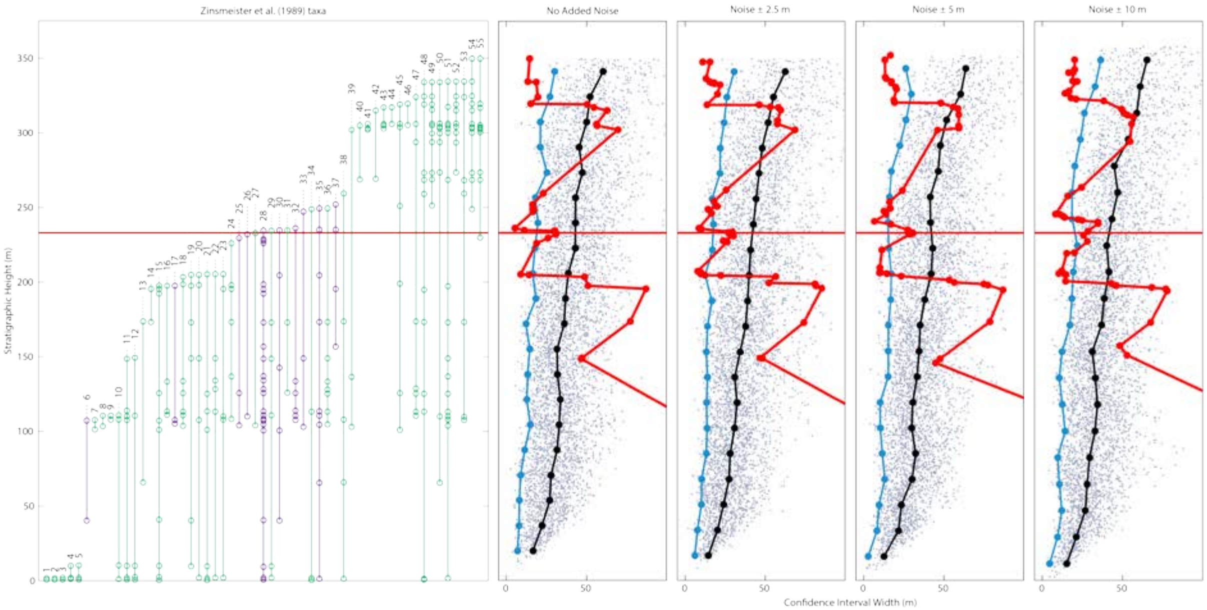

Matlab Codes

Confint.m

```
%Script used for Tobin paper on Confidence Intervals of Mass Extinctions
%Uses basic ideas from Wang & Marshall (2004) but applies them iteratively
%to data set, starting with first two taxa, then adding one at a time until
%all are included
%Relies on taxaplot.m - written by Tobin, makes plot of species occurrences
%Relies on multispec.m - written by Tobin, performs iterative
%Wang&Marshall(2004) style analysis to full data set, returns data
%In some configurations, relies on randfixedsum.m downloaded from mathworks
%Currently data must be loaded in text files of the exact right format

clear all
close all
opengl software
%Loads main data file,
%column 1 must be stratigraphic height, column 2  must be species number
%There are 4 places where the input file names must be replaced
load Witts2016A.txt
strat=Witts2016A(:,1);

%Adds random noise to stratigraphic heights, must be calibrated manually
% If set to 0, no added noise
jitmod=0;
jit=jitmod*rand([length(strat),1]);
jit=jit-(mean(jit));
strat=strat+jit;

spec=Witts2016A(:,2);
species=unique(spec);

%Loads species names file, column 1 is species name, column 2 is type of
%organism, currently 'benthic' or 'planktic' or 'misc' are acceptable
%Should be in order of the numerical species numbers in above file
t=textread('Witts2016ASpecies.txt','%s'); %Currently can't handle spaces in
input file, replace  all with '_'
speciesnames=t(1:2:length(t));
speciestype=t(2:2:length(t));
for i=1:length(speciesnames)
    speciesnames(i)=strrep(speciesnames(i),'___',' ');
    speciesnames(i)=strrep(speciesnames(i),'__',' ');
    speciesnames(i)=strrep(speciesnames(i),'_',' ');
end

% %If any species has 0 occurrences, this section removes them
% hu=1:max(species);
% no_occ=setdiff(hu,species);
% %species1=1:max(species)-length(no_occ);
% speciesnames(no_occ)=[];
% speciestype(no_occ)=[];
% for i=1:length(species)
```

```
%     repl=find(spec==species(i));
%     spec(repl)=i;
%     species(i)=i;
% end


%%%% OPTIONS %%%%%

%Use last LAD for base confidence interval (see Wang & Marshall 2004)
uselast=0; % Left a 0 for analysis in manuscript

%Remove singleton taxa 1=remove
sing=1; %Must be left at 0, otherwise statistical techniques cannot apply

%Remove taxa labelled miscellaneous 1=remove
usemisc=1;

%Confidence threshold for multi species extinction confidence intervals
%(0-1). Set as 0.95 for manuscript
conf_thresh=0.95;

%When plotting with taxaplot, whether to use taxa names (1), or to use just
%numbers to represent taxa (0)
%Will print out numbers and taxa in matlab main screen
usenames=0;

% Number of random data sets that are generated for background comparison
runs=100; %Set to 100 for most analysis in manuscript

%Use log based error calculations for randomized background data?
%Most of this data is not normally distributed, if use_log=1 then values
%are converted to log space, error bars calculated, then converted back
%Doesn't actually matter to figures used in publication, only those with
%error bars not percentiles as comparison
uselog=0;

%Asks for type of sorting FA=First appearance LA=Last Appearance
%LA will also trigger confidence interval testing (eventually)
sortby=2; %input('Sort by? 1-Original 2-LA 3-FA   ');

%Asks for confidence intervals for plotting, does not affect how
%multispec.m functions in calculating confidence intervals
conf=(input('Add confidence interval to ranges? 0-99%   '))/100;

%Asks for number of bins for richness and calculations (uses confidence
%interval inputted above
%Also used in calculating 2.5% percentile and medians on random data
numbins=(input('How many bins?   '));

%Asks for 'known' location of extinction boundary if present
%Non-numeric answers lead to no plotting
%Not used in calculation
exbound=(input('Stratigraphic level of extinction boundary (or 0)?   '));

```

```
ben_ind=strmatch('benthic',speciestype);
pla_ind=strmatch('planktic',speciestype);
misc_ind=strmatch('misc',speciestype);
% misc_ind=find(ismember(species,misc_inde)==1);
%
% %Removes miscellaneous organisms
% if usemisc==1
%     species(misc_ind,:)=[];
%     speciesnames(misc_ind,:)=[];
%     speciestype(misc_ind,:)=[];
% else
% end


%Remove singleton taxa
if sing==1
    j=1;
    singles=[];
    for i=1:max(spec)
        k=find(spec==i);
        if length(k)<2
            singles(j)=i;
            j=j+1;
        else
        end

    end
    singles=singles';
    x=ismember(species,singles);
    c=find(x==1);
    speciesnames(c,:)=[];
    species(c,:)=[];
    speciestype(c,:)=[];
else
end


%Makes second matrix for Wang 2012 processing
wangmat=NaN(length(species),5);

%Makes each row in specmat one species with all occurrences
%Also completes wangmat with species, fad, lad, range, occurrences
occurr=cell(length(species),1);
for i=1:length(species)
    m=species(i);
    k=find(spec==m);
    j=strat(k);
    maxj=max(j);
    minj=min(j);
    ranj=maxj-minj;
    wangmat(i,1)=species(i);
    wangmat(i,2)=minj;
    wangmat(i,3)=maxj;
    wangmat(i,4)=ranj;
    wangmat(i,5)=length(j);
    occurr{i}=sort(j);
```

```
end

%Runs taxaplot which displays the species occurrences in stratigraphic
%context
[sortwang,sortind,sortupper]=taxaplot(wangmat,occurr,speciesnames,speciestype
,sortby,conf,exbound,usenames,1);
allspectext=[];
if usenames==0
    speciesorder=speciesnames(sortind);
    colheadings={'Taxon name'};
    for rw=1:length(sortind);
        rowheadings{rw}=[num2str(rw)];
        eachspec=strcat(num2str(rw),'-',speciesnames(sortind(rw)),';');
        allspectext = [allspectext,eachspec];
    end
    figcap=strjoin(allspectext);
    val=cellfun(@(x) numel(x),speciesnames);
    displaytable(speciesorder,colheadings,max(val),{},rowheadings)

else
end


[usewidth,usebase,usetop,h1]=multispec(wangmat,conf_thresh,uselast);

%     Turned this figure off because it's much better displayed by figure 3
%     figure(2)
%     title('Base and Top of Multi-species Extinction Confidence Intervals')
%     hold on
%     plot(usebase,h,'o')
%     plot(usetop,h,'o')
%     if exbound==0
%     else
%         hline=refline(0,exbound);
%         set(hline,'Color','r')
%     end

figure(3)
title('Multi-species Extinction Confidence Interval Width')
hold on
plot(usewidth,h1,'-bo','MarkerFaceColor','b','MarkerEdgeColor','none')
if exbound==0
else
    hline=refline(0,exbound);
    set(hline,'Color','r','LineWidth',2)
end


RichOrigExt(wangmat,conf,numbins,exbound,[4 5 6])


%%%%%%%%%%
%%% This routine tries to generate confidence intervals from random fossil
%%% distributions based on the original distribution
%%%%%%%%%%%%%%%%
```

```
%Makes second matrix for Wang 2012 processing

dens_wid=[];
dens_h=[];


for i=1:runs
wangmatr=NaN(length(species),5);

%This IF section either completely randomizes species for, or leaves the
%distribution of species occurrences the same, but randomizes their
%positions
randomspecies=1; %randomspecies=1, then species completely randomized

%Percent survival of randomized species
%Allows for random ammounts of species to go extinct
%Can change these thresholds, has range within []
%Added a few lines to make percent survival based on estimate of percent
%survival in real data
%Surival percent calculated using the user inputted conf, and finding how
%many overlap with highest LAD, this process could probably be refined a
%bit
confupper=sortupper+wangmat(:,3);
survivalpoint=max(wangmat(:,3));
survivors=find(confupper>=survivalpoint);
real_surv_perc=length(survivors)/length(wangmat(:,1));
real_surv_low=real_surv_perc-0.10;
real_surv_high=real_surv_perc+0.10;
if real_surv_high>1
    real_surv_high=1;
else
end
if real_surv_low<0
    real_surv_low=0;
else
end

surv_perc_rang=round(100.*[real_surv_low,real_surv_high]);
%surv_perc_rang=[100 100];
rand_percent_surv=randi(surv_perc_rang,1)/100;
num_ext=round((1-rand_percent_surv)*length(species));
num_surv=round(rand_percent_surv*length(species));
nn=randi([round(min(wangmat(:,3))), round(max(wangmat(:,3)))],num_ext,1);
tt=(ones(num_surv,1))*max(strat);
ran_LAD=vertcat(nn,tt);
ran_LAD=ran_LAD(randperm(length(ran_LAD)));%Above this is building ran_LAD -
random LADs to be used in generating random species ranges
min_FAD=min(wangmat(:,2));
max_LAD=max(wangmat(:,3));
specr=[];
stratr=[];
speciesr=1:length(species);

%Generates specr and stratr (randomized, but analagous to spec and strat)
%In this for loop, each species maintains the same number of occurrences
```

```
% for pl=1:(length(species))
%     uy=species(pl);
%     k=find(spec==uy);
%     initlength=length(specr);
%     p=(rand(length(k),1).*((ran_LAD(pl))-min_FAD))+min_FAD;
%     specr((initlength+1):(initlength+length(k)))=repmat(pl,length(p),1);
%     stratr((initlength+1):(initlength+length(k)))=p;
%
% end

%Generates specr and stratr
%In this loop, the number of occurrences per species is randomized, as are
%the stratigraphc levels

%stratr=((max_LAD-min_FAD).*rand(length(strat)-length(singles),1))+min_FAD;
tempspec=round(randfixedsum(length(speciesr),1,length(strat)-
length(singles),2,max(wangmat(:,5))));
rounddiff=sum(tempspec)-(length(strat)-length(singles));
[Y,I]=max(tempspec);
tempspec(I)=Y-rounddiff;
for ht=1:length(species)
    uy=species(ht);
    k=find(spec==uy);
    reps=ht.*ones(tempspec(ht),1);
    specr=vertcat(specr,reps);
    p=(rand(length(reps),1).*((ran_LAD(ht))-min_FAD))+min_FAD;
    stratr=vertcat(stratr,p);

end


%Makes each row in wangmat one species with all occurrences
%Also completes wangmat with species, fad, lad, range, occurrences
%This time wangmatr is randomized based on specr and stratr, instead of
%using spec and strat real data
occurr_r=cell(length(speciesr),1);
for i=1:length(speciesr)
    m=speciesr(i);
    k=find(specr==m);
    j=stratr(k);
    maxj=max(j);
    minj=min(j);
    ranj=maxj-minj;
    wangmatr(i,1)=speciesr(i);
    wangmatr(i,2)=minj;
    wangmatr(i,3)=maxj;
    wangmatr(i,4)=ranj;
    wangmatr(i,5)=length(j);
    occurr_r{i}=sort(j);
end

%Sorts wangmatr by preferred FAD,LAD,Original order, at this point, won't
%work/matter unless sorted by LAD
if sortby==1
    sortwangr=wangmatr;
    sortindr=1:length(wangmatr);
```

```
elseif sortby==2
    [sortwangr,sortindr]=sortrows(wangmatr, [3 -5  2]);
elseif sortby==3
    [sortwangr,sortindr]=sortrows(wangmatr, [2 -5  3]);
end

%Sorts non-wangmat variables by same index
sortoccurrr=occurr_r(sortindr);
sortnamesr=speciesnames(sortindr);
sorttypesr=speciestype(sortindr);
sortspeciesr=species(sortindr);

[usewidthr,usebaser,usetopr,hr]=multispec(wangmatr,conf_thresh,uselast);

figure(7)
hold on
plot(usewidthr,hr,'-o')
if exbound==0
else
    hline=refline(0,exbound);
    set(hline,'Color','r','LineWidth',2)
end
% ylim([min(h) max(h)])
% xlim([min(usewidth) max(usewidth)])

%Collects all data in figure 7 for use in plotting density or similar
dens_wid=vertcat(dens_wid,usewidthr);
dens_h=vertcat(dens_h,hr);


end

figure(7)
hold on
fsd=plot(usewidth,h1,'-
ro','LineWidth',2,'LineSmoothing','off','MarkerSize',6,'MarkerFaceColor','r',
'MarkerEdgeColor','none');
uistack(fsd,'top')
legend('Random Trials','Fossil Data','Location','Southeast')
w= get(get(figure(7),'CurrentAxes'),'XLim');
xlim([w(2)/-20 w(2)]);
if exbound==0
else
    hline=refline(0,exbound);
    set(hline,'Color','r','LineWidth',2)
end

%Removes dens_ values that are NaN
remo=find(isnan(dens_wid)==1);
dens_wid(remo)=[];
dens_h(remo)=[];


[dens_h_s,dens_ind]=sort(dens_h);
dens_wid_s=dens_wid(dens_ind);
```

```
%Creates stratigraphic bins for binning and averaging confidence interval
%widths
inte=(max(dens_h)-min(dens_h))/numbins;
dens_bins=min(dens_h):inte:max(dens_h);
rounded_h=interp1(dens_bins,dens_bins,dens_h,'nearest');

%Creates stratigraphic bins and their mean/sd from data points in figure 7
for jt=1:numbins
    hr= find(dens_h>=dens_bins(jt) & dens_h<=dens_bins(jt+1));
    lp=dens_wid(hr);
    dens_val(jt)=mean(lp);
    dens_sd(jt)=std(lp);
    dens_val_log(jt)=mean(log(lp));
    dens_val_log_sd(jt)=std(log(lp));
    dens_cent(jt)=mean([dens_bins(jt) dens_bins(jt+1)]);
    dens_025(jt)=prctile(lp,2.5);
    dens_975(jt)=prctile(lp,97.5);
    dens_med(jt)=median(lp);
end

%This figure plots the bins generated from random simulations and the
confidence
%interval widths from the fossil data to see if fossil data is outside
%random noise
figure(8)
dots=plot(dens_wid,dens_h,'.','MarkerSize',5,'Color',[160 168 193]./255);
%boxes=boxplot(dens_wid,rounded_h,'Orientation','horizontal');
%Tried boxplot, realized all it does is hide information and make plotting
%hard
hold on

%Sets the lowerbound on error bars to 0, since negative stratigraphic
%widths are impossible
lowerror=dens_sd*2;
lowbound=dens_val-lowerror;
toolow=find(lowbound<0);
lowerror(toolow)=lowerror(toolow)+lowbound(toolow);
higherror=dens_sd*2;

%Generates error bounds from log data if uselog=1
uperr=exp(dens_val_log+(dens_val_log_sd*2))-dens_val;
downerr=exp(dens_val_log-(dens_val_log_sd*2))-dens_val;
loglow=find(downerr<0);
%downerr(loglow)=downerr(loglow)+(dens_val(loglow)-downerr(loglow));
if uselog==1
    lowerror=downerr;
    higherror=uperr;
else
end

errorbound=herrorbar(dens_val,dens_cent,lowerror,higherror,'.b');
set(errorbound,'MarkerSize',1,'LineWidth',2);
fossdata=plot(usewidth,h1,'-
ro','LineWidth',3,'MarkerSize',8,'MarkerEdgeColor','none','MarkerFaceColor','
r','LineSmoothing','off');
```

```
if exbound==0
else
    hline=refline(0,exbound);
    set(hline,'Color','r','LineWidth',2)
end
% xlabel('Confidence Interval Width (m)')
% ylabel('Stratigraphic Height (m)')
 legend([dots fossdata errorbound(1,:)],'Random Trials','Fossil Data','Error
Bounds','Location','Southeast')
%Plots an example fossil stratigraphy (the last one generated) for inspection

figure(2)
dots=plot(dens_wid,dens_h,'.','MarkerSize',5,'Color',[160 168 193]./255);
hold on
pct025=plot(dens_025,dens_cent,'-ob','LineWidth',2,'MarkerFaceColor','b');
%pct975=plot(dens_975,dens_cent,'-ob','LineWidth',2,'MarkerFaceColor','b');
meds=plot(dens_med,dens_cent,'-ok','LineWidth',2,'MarkerFaceColor','k');
fossdata=plot((usewidth),h1,'-
ro','LineWidth',3,'MarkerSize',8,'MarkerEdgeColor','none','MarkerFaceColor','
r','LineSmoothing','off');
%finds max values of important data for better plotting later
max_med=max(dens_med);
max_foss=max(usewidth);

dlx=get(get(figure(2),'CurrentAxes'),'XLim');
set(get(figure(2),'CurrentAxes'),'XLim',[dlx(2)/-20 dlx(2)])
if exbound==0
else
    hline=refline(0,exbound);
    set(hline,'Color','r','LineWidth',2)
end
taxaplot(wangmatr,occurr_r,speciesnames,speciestype,sortby,conf,exbound,usena
mes,9);
figure(9)
title(['Simulated Random Data Set: ',num2str(100*rand_percent_surv),'%
Survival ','(Range used: ',num2str(round(100*real_surv_low)),'% -
',num2str(round(100*real_surv_high)),'%)'])



figure(10)
h(101)=subplot(1,2,1);
h(102)=subplot(1,2,2);
c=figure(1);
k=figure(3);
copyobj(allchild(get(c,'CurrentAxes')),h(101));
copyobj(allchild(get(k,'CurrentAxes')),h(102));
linkaxes([h(101),h(102)],'y')
set(h(102),'YTick',[]);
set(h(102),'Position',[0.64 0.11 0.2 0.815]);
set(h(101),'Position',[0.13 0.11 0.51 0.815]);
set(h(101),'XTick',[]);
xlabel(h(102),'Confidence Interval Width (m)');
```

```
figure(11)
h(111)=subplot(1,2,1);
h(112)=subplot(1,2,2);
d=figure(7);
m=figure(2);
copyobj(allchild(get(d,'CurrentAxes')),h(111));
copyobj(allchild(get(m,'CurrentAxes')),h(112));
linkaxes([h(111), h(112)]);
%axis(h(111),'tight');
set(h(112),'YTick',[]);
b=get(h(111),'XLim');
set(h(111),'XLim',[b(2)/-20 b(2)]);
xlabel(h(111),'Confidence Interval Width (m)')
xlabel(h(112),'Confidence Interval Width (m)')
ylabel(h(111),'Stratigraphic Height (m)')
figure(11)
suptitle(strcat(num2str(runs),' random trials'))
if exbound==0
else
    hline=refline(0,exbound);
    set(hline,'Color','r','LineWidth',2)
end
legend(h(112),[dots fossdata errorbound(1,:)],'Random Trials','Fossil
Data','Error Bounds','Location','Southeast')
legend(h(111),'Random Trials','Fossil Data','Location','Southeast')

figure(12)
h(121)=subplot(1,2,1);
h(122)=subplot(1,2,2);
suptitle(strcat(num2str(runs),' random trials'))
copyobj(allchild(get(d,'CurrentAxes')),h(121));
copyobj(allchild(get(m,'CurrentAxes')),h(122));
linkaxes([h(121), h(122)]);
%axis(h(111),'tight');
b=get(h(111),'XLim');
set(h(111),'XLim',[b(2)/-20 b(2)]);
plx=get(get(figure(3),'CurrentAxes'),'XLim');
ply=get(get(figure(3),'CurrentAxes'),'YLim');
set(h(121),'XLim',[plx(2)/-20 plx(2)]);
set(h(121),'YLim',ply);
set(h(122),'YTick',[]);
xlabel(h(121),'Confidence Interval Width (m)')
xlabel(h(122),'Confidence Interval Width (m)')
ylabel(h(121),'Stratigraphic Height (m)')
legend(h(122),[dots fossdata errorbound(1,:)],'Random Trials','Fossil
Data','Error Bounds','Location','Southeast')
legend(h(121),'Random Trials','Fossil Data','Location','Southeast')

figure(13)
h(131)=subplot(1,2,1);
box on
h(132)=subplot(1,2,2);
box on
c=figure(1);
k=figure(8);
copyobj(allchild(get(c,'CurrentAxes')),h(131));
```

```
copyobj(allchild(get(k,'CurrentAxes')),h(132));
linkaxes([h(131),h(132)],'y')
set(h(132),'YTick',[]);
set(h(132),'Position',[0.64 0.11 0.2 0.815]);
set(h(131),'Position',[0.13 0.11 0.51 0.815]);
set(h(131),'XTick',[]);
xlabel(h(132),'Confidence Interval Width (m)');
set(h(132),'XLim',[plx(2)/-20 plx(2)]);


figure(14)

h(141)=subplot(1,2,1);
%box on
h(142)=subplot(1,2,2);
box on
c=figure(1);
k=figure(2);
copyobj(allchild(get(c,'CurrentAxes')),h(141));
copyobj(allchild(get(k,'CurrentAxes')),h(142));
glx=get(get(figure(8),'CurrentAxes'),'XLim');
linkaxes([h(141),h(142)],'y')
set(h(142),'YTick',[]);
set(h(142),'Position',[0.64 0.11 0.2 0.815]);
set(h(141),'Position',[0.13 0.11 0.51 0.815]);
set(h(141),'XTick',[]);
xlabel(h(142),'Confidence Interval Width (m)');


%set(h(142),'XLim',[glx(2)/-20 glx(2)]);
max_use=max(max_med,max_foss);
set(h(142),'XLim',[max_use/-20 max_use*1.1])
if exbound==0
else
    hline=refline(0,exbound);
    set(hline,'Color','r','LineWidth',2)
end
% set(gca,'Color',[0 0 0]);
% legend('Trials','2.5%','Med.','Foss.','Location','southeast')

% set(findall(figure(14),'-property','FontColor'),'FontColor','w')

%Changes standard pure blue and red to something less harsh
myblue=[30 144 205]./255;
myred=[1 0 0]; %[158 27 50]./255;
mygrey=[150 158 163]./255;
%Changes to all figures to make prettier
for i=1:14
    set(findall(figure(i),'-property','FontName'),'FontName','MyriadPro-
Regular') %Changes font type
    set(findall(figure(i),'Color','b'),'Color',myblue) %Changes all raw blue
to another color
    set(findall(figure(i),'MarkerFaceColor','b'),'MarkerFaceColor',myblue)
%MarkerFaceColor is apparently a different property than Color
    set(findall(figure(i),'Color','r'),'Color',myred) %Changes red
```

```
    set(findall(figure(i),'MarkerFaceColor','r'),'MarkerFaceColor',myred)
    set(findall(figure(i),'-property','FontSize'),'FontSize',20) %Changes
font size


end

%Abandoned code, creates 97.5-2.5% range error bars in a rolling window,
%doesn't really add any useful information, looks messy

% wind=401;
% [dens_h_s,h_ind]=sort(dens_h_s);
% dens_wid_s=dens_wid_s(h_ind);
% for i=(wind/2+.5):(length(dens_wid_s)-(wind/2-.5))
%     int=(i-(wind/2-0.5)):(i+(wind/2-0.5));
%
%     h_h(i-(wind/2-0.5))=mean(dens_h_s(int));
%     d_med(i-(wind/2-0.5))=median(dens_wid_s(int));
%     p025(i-(wind/2-0.5))=prctile(dens_wid_s(int),2.5);
%     p975(i-(wind/2-0.5))=prctile(dens_wid_s(int),97.5);
%
% end
%
% plot(d_med,h_h)
% hold on
% dots=plot(dens_wid,dens_h,'.','MarkerSize',5,'Color',[160 168 193]./255);
% uistack(dots,'bottom')
% plot(p025,h_h)
% plot(p975,h_h)



opengl hardware

%figures to close
figcl=[4 5 6];%[3 4 5 6 8 9 10 11 12 13]
for i=figcl
close(figure(i))
end


load gong
sound(y,Fs)
```

multispec.m

```
function[usewidth,usebase,usetop,h]=multispec(wangmat,conf_thresh,uselast)

%First loops by LAD of fossils, starting from lowest and including
%increasing numbers of species
%For each set of included species, a variety of individual species confidence
%intervals are attempted to find minimum width mass extinction confidence
%interval - uses methods from Wang & Marshall 2004
%

%wangmat is a matrix consisting of 5 columns
    %column 1 is just a numeric species assignment (1-max)
    %column 2 is the stratigraphic height of the FAD for the species
    %column 3 is the stratigraphic height of the LAD for the species
    %column 4 is the stratigraphic range for the species
    %column 5 is the number of occurrences for that species

%Confthresh (0-1) is the threshold for which the multispecies extinction
%interval needs to reach before it is accepted

%uselast (0,1) determines whether to use the highest LAD as the
%stratigraphic base of the confidence interval


    h=unique(wangmat(:,3));

    %Preallocation
    wconfwidth=cell(length(h),1);
    wconfbase=cell(length(h),1);
    wconftop=cell(length(h),1);
    wincspecies=cell(length(h),1);
    wincind=cell(length(h),1);
    wconftest=cell(length(h),1);

    usewidth=NaN(length(h),1);
    usetop=NaN(length(h),1);
    usebase=NaN(length(h),1);
    useincspecies=cell(length(h),1);
    useincind=cell(length(h),1);
    useconf=NaN(length(h),1);
    usenum=NaN(length(h),1);
    %End Preallocation


    %overall loop by last occurrence
    for wn=2:length(h)
        f=find(wangmat(:,3)>h(wn));
        twang=wangmat;
        twang(f,:)=[];
```

```
        %Builds testing confidence intervals
        conflow=1/length(twang(:,1));
        conftest=(conflow:conflow:0.50);
        %incspecies=NaN(1,length(conftest));
        incspecnum=NaN(1,length(conftest));
        for j=1:length(conftest) %loops different confidence testing intervals (within last occurrence loop)

            %Preallocation (within loop)
%             confwidth=NaN(length(conftest),1);
%             confbase=NaN(length(conftest),1);
%             conftop=NaN(length(conftest),1);
%             incspecies=cell(length(conftest),1);
%             incind=cell(length(conftest),1);
            %End Preallocation (within loop)

            %Makes confext variable - amount to extend ranges based on size of
            %conftest
            confext=NaN(length(twang(:,1)),1);
            for g=1:length(twang(:,1))
                confext(g)=((1-conftest(j))^(-1/twang(g,5))-1)*(twang(g,4));
            end
            testupper=twang(:,3)+confext;
            testwang=[twang testupper];
            [testsort,testind]=sortrows(testwang, [-6 2 -5]);
            t=length(testind);

%             testoccurr=occurr(testind);
%             testnames=speciesnames(testind);
%             testtypes=speciestype(testind);
%             testspecies=species(testind);

            %Creates binomial probability distributions as in Wang 2004
            y=1:length(testind);%(wangmat(:,1));
            bindist=binopdf(y,length(testind),conftest(j));
            [sortbindist,bin_ind]=sort(bindist,'descend');


            %Counts species until 95% multi species confidence interval is reached

            for i=1:length(bindist)
                p=sum(sortbindist(1:i));

                if p>conf_thresh
                    incspecnum(j)=i; %number of species needed to reach 0.95
                    incspecies{j}=sortbindist(1:i); %which binomial dists are included
                    incind{j}=bin_ind; %full index of which species
                    [lowest,least]=min(incspecnum);
                    q=incind{least}(1:lowest);
                    w=testsort(q,:);
```

```
                    conftop(j)=max(w(:,6)); %top of confidence interval once
95% is reached
                    if uselast==1
                        confbase(j)=h(wn);
                        confwidth(j)=conftop(j)-h(wn);
                    else
                        confbase(j)=min(w(:,6)); %base of confidence interval
once 95% is reached
                        confwidth(j)=range(w(:,6));
                    end
                    break
                elseif p<conf_thresh && i==length(bindist) %if 95% never
reached, return NaN
                    confwidth(j)=NaN;
                    confbase(j)=NaN;
                    conftop(j)=NaN;
                    incspecies{j}=NaN;
                    incind{j}=NaN;
                end
            end

        end

        %Creates cell arrays of all the
        wconfwidth{wn}=confwidth;
        wconfbase{wn}=confbase;
        wconftop{wn}=conftop;
        wincspecies{wn}=incspecies;
        wincind{wn}=incind;
        wconftest{wn}=conftest;
    end

    %Finds minimum width confidence interval for each group of species
    %included
    for ind=2:length(h)
        [~, minind]=min(wconfwidth{ind});
        usewidth(ind)=wconfwidth{ind}(minind);
        usetop(ind)=wconftop{ind}(minind);
        usebase(ind)=wconfbase{ind}(minind);
        useincspecies(ind)=wincspecies{ind}(minind);
        useincind(ind)=wincind{ind}(minind);
        useconf(ind)=wconftest{ind}(minind);
        usenum(ind)=length(useincind{ind});

    end

end
```

taxaplot.m

```
function [ sortwang,sortind,sortupper ] =
taxaplot(wangmat,occurr,speciesnames,speciestype,sortby,conf,exbound,usenames
,fignum)
%Plots taxa range chart according to previous sorting

%wangmat is a matrix consisting of 5 columns
    %column 1 is just a numeric species assignment (1-max)
    %column 2 is the stratigraphic height of the FAD for the species
    %column 3 is the stratigraphic height of the LAD for the species
    %column 4 is the stratigraphic range for the species
    %column 5 is the number of occurrences for that species

%occurr is formatted as cell, where each cell is a list of the species
%occurrences

%speciesnames is a list of species names , in the same order as the species
%in wangmat

%speciestype is a list of species types, in the same order as the species
%in wangmat
    %Currently acceptable options are 'planktic' 'benthic' 'misc'
    %Misc are excluded, planktic plotted as red, benthic plotted as blue

%sortby = 1,2,3 options
    %1 = original order
    %2 = LAD, then by FAD, then by number of occur
    %3 = FAD, then by LAD, then by number of occurr

%conf is a percentage (0-1) for how much of a single species confidence
%interval is added to the graphic display, not used in calculations
%otherwise

%exbound is the stratigraphic height of a knwown extinction boundary

%Fignum is the number of a figure to be displayed, necessary if taxaplot is
%run twice within the same script


%Sorts matrix by occurrences as desired

species=wangmat(:,1);
if sortby==1
    sortwang=wangmat;
    sortind=1:length(wangmat);
elseif sortby==2
    [sortwang,sortind]=sortrows(wangmat, [3 -5  2]);
elseif sortby==3
    [sortwang,sortind]=sortrows(wangmat, [2 -5  3]);
end

%Sorts non-wangmat variables by same index
```

```
sortoccurr=occurr(sortind);
sortnames=speciesnames(sortind);
sorttypes=speciestype(sortind);
sortspecies=species(sortind);


%Tex strings end up with generating warning, this suppresses those warnings
%warning('off','MATLAB:gui:latexsup:BadTeXString');

%Depending on scale, adjustheight adds some envelope to beginning of
%species labelling, currently scaled to max LAD
adjustheight=(max(wangmat(:,3))-min(wangmat(:,2)))*0.01;


%This for loop generates plot of taxon ranges with desired confidence
%intervals
sortupper=NaN(length(sortwang),1);

        for i=1:length(sortwang)
            figure(fignum)
            hold on
            sortupper(i)=((1-conf)^(-1/sortwang(i,5))-1)*(sortwang(i,4));
            %Sets no confidence interval (1 sample) to 0 rather than NaN
            if isnan(sortupper(i))==1
                sortupper(i)=0;
            end


            xs=ones(sortwang(i,5),1);
            xs=xs.*i;



            if strmatch('benthic',sorttypes(i))==1

                plot(xs,sortoccurr{i},'-o','LineWidth',1)
                h=errorbar(xs(end),sortwang(i,3),0,sortupper(i));
            elseif strmatch('planktic',sorttypes(i))==1

                plot(xs,sortoccurr{i},'-ro','LineWidth',1)
                h=errorbar(xs(end),sortwang(i,3),0,sortupper(i),'r');
            end

            %The following is the code from removetees.m
            dataLen = length( get(h, 'xdata') );
            candidateList = findall(h);
            for candidate = candidateList(:)'
                candLen = length( get(candidate, 'xdata') );
                if candLen == 9 * dataLen
                xOrg = get(candidate, 'xdata');
                yOrg = get(candidate, 'ydata');
                valuesToExtract = find( kron( ones(1,dataLen), [ones(1,3)
zeros(1,6)]) );
                xNew = xOrg(valuesToExtract);
```

```
                yNew = yOrg(valuesToExtract);
                set(candidate, 'xdata', xNew);
                set(candidate, 'ydata', yNew);
                end
            end
            %ends removetees.m code

            hChildren = get(h, 'children');
            set(hChildren(2), 'linestyle',':')

            if usenames==1


set(text(xs(end),sortwang(i,3)+sortupper(i)+adjustheight,sortnames(i)),'Rotat
ion',90)
            else

set(text(xs(end),sortwang(i,3)+sortupper(i)+adjustheight,num2str(i)),'Rotatio
n',90)
            end



        end




        ylabel('Stratigraphic Height (m)')
        set(gca,'XTick',[])
        tem=get(gca,'XLim');
        set(gca,'XLim',[0 tem(2)+2]);

                if exbound==0
                else
                    hline=refline(0,exbound);
                    set(hline,'Color','r','LineWidth',2)
                end
end
```
